# Spatial and ecological population genetic structures within two island‐endemic *Aeonium* species of different niche width

**DOI:** 10.1002/ece3.1682

**Published:** 2015-09-14

**Authors:** David E. V. Harter, Mike Thiv, Alfons Weig, Anke Jentsch, Carl Beierkuhnlein

**Affiliations:** ^1^ BiogeographyBayCEERUniversity of BayreuthBayreuthGermany; ^2^ State Museum of Natural History StuttgartStuttgartGermany; ^3^ DNA Analytics and EcoinformaticsBayCEERUniversity of BayreuthBayreuthGermany; ^4^ Disturbance EcologyBayCEERUniversity of BayreuthBayreuthGermany

**Keywords:** Gene flow barriers, island biogeography, isolation by distance, isolation by ecology, landscape genetics, niche width

## Abstract

The Crassulacean genus *Aeonium* is a well‐known example for plant species radiation on oceanic archipelagos. However, while allopatric speciation among islands is documented for this genus, the role of intra‐island speciation due to population divergence by topographical isolation or ecological heterogeneity has not yet been addressed. The aim of this study was to investigate intraspecific genetic structures and to identify spatial and ecological drivers of genetic population differentiation on the island scale. We analyzed inter simple sequence repeat variation within two island‐endemic *Aeonium* species of La Palma: one widespread generalist that covers a large variety of different habitat types (*Ae. davidbramwellii*) and one narrow ecological specialist (*Ae. nobile*), in order to assess evolutionary potentials on this island. Gene pool differentiation and genetic diversity patterns were associated with major landscape structures in both species, with phylogeographic implications. However, overall levels of genetic differentiation were low. For the generalist species, outlier loci detection and loci–environment correlation approaches indicated moderate signatures of divergent selection pressures linked to temperature and precipitation variables, while the specialist species missed such patterns. Our data point to incipient differentiation among populations, emphasizing that ecological heterogeneity and topographical structuring within the small scales of an island can foster evolutionary processes. Very likely, such processes have contributed to the radiation of *Aeonium* on the Canary Islands. There is also support for different evolutionary mechanisms between generalist and specialist species.

## Introduction

Species radiations on oceanic archipelagos provide illustrative showcases of evolutionary patterns (see, e.g., Baldwin et al. 1999; Losos and Ricklefs 2009 and references therein), which have been motivating fundamental phylogenetic, biogeographical, and ecological research (e.g., Lösch 1990; Baldwin and Sanderson 1998; Gillespie 2004; Grant and Grant 2006). However, there are still open questions regarding processes and drivers. It is unclear in many cases whether and how often species have diverged in allopatry among islands (isolated evolution after colonization) or whether within‐island speciation processes have contributed to such radiations (e.g., Whittaker and Fernández‐Palacios 2007; Losos and Ricklefs 2009; Thiv et al. 2010). While past allopatric speciation among islands is relatively straightforward to infer from phylogenetic and biogeographical patterns, detections of intra‐island evolutionary divergence additionally require the demonstration of evolutionary divergent forces on the comparably small scales of an island (see, e.g., Savolainen et al. 2006; Mallet et al. 2014; Papadopulos et al. 2014; Suárez et al. 2014).

Speciation can be initiated by spatial or ecological isolation among populations. Spatial isolation limits gene flow, so that genetic drift and divergent natural selection can subsequently lead to gene pool divergence (Wright 1943; Slatkin 1993; Hutchison and Templeton 1999). In ecological speciation, strong divergent selection drives populations to differential adaptations, resulting in reciprocal maladaptation and gradual built‐up of reproductive isolation by selection against migrants (Nosil 2012; Wang and Bradburd 2014). Further on, interactions between spatial and ecological mechanisms can easily affect the formation of reproductive barriers between populations and subsequent species divergence (Rundell and Price 2009; Nosil 2012; Orsini et al. 2013; Wang et al. 2013).

Differences in selection regimes among populations due to environmental gradients and heterogeneity are potent and common drivers of evolutionary divergence and speciation (Doebeli and Dieckmann 2003; Nosil 2012; Stein and Kreft 2015). In island biogeographical theory, strong environmental heterogeneity of an island (e.g., climatic, geological, or edaphic structuring) and also spatial factors such as area and topographical dissection are often postulated to facilitate speciation (Stuessy 2007; Whittaker et al. 2008; Losos and Ricklefs 2009; Vitales et al. 2014). However, comparably few studies directly assessed and quantified these evolutionary effects on the population level within single islands (but see Mallet et al. 2014; Papadopulos et al. 2014; Stacy et al. 2014; Suárez et al. 2014).

The Crassulacean genus *Aeonium* is an iconic example of plant species radiations on islands. On the Canarian archipelago, it comprises about 28 species plus a series of distinct subspecies (Liu 1989; Arechavaleta Hernández et al. 2010; numbers vary between authors and taxonomic treatments). The genus was shown to have evolved on the Canaries (Mes et al. 1996; Mort et al. 2002), with a relatively young phylogenetic origin in the late Miocene (Kim et al. 2008) or even later (Thiv et al. 2010). A large variety of ecological niches, morphological forms, and ecophysiological characteristics (Liu 1989; Lösch 1990; Mort et al. 2007) suggests adaptive speciation processes in the history of *Aeonium* (Lems 1960; Jorgensen and Frydenberg 1999; Jorgensen 2002; Thiv et al. 2010). However, the spatial scales of these processes, *that is,* if species divergences occurred within single islands or only between islands, and the evolutionary drivers of differentiation have not been resolved.

Probably, inter‐island allopatric speciation has played a major role for the radiation of *Aeonium* (Mes and Hart 1996; Thiv et al. 2010). Nevertheless, intra‐island events cannot be ignored and are suggested, *for example,* for the closely related Tenerife endemics *Ae. haworthii*,* Ae. urbicum,* and *Ae. pseudourbicum* (Liu 1989; Jorgensen 2002; Mort et al. 2002), and are also possible for *Ae. canariense*,* Ae. tabuliforme,* and *Ae. cuneatum* on the same island (Liu 1989). Nevertheless, the long and vivid geological history of Tenerife that integrated three former distinct islands into one (Carracedo et al. 2002) makes it difficult to separate between‐ and within‐island speciation processes on this major island.

In contrast, La Palma is much younger (maximum age: 1.77 Ma) than Tenerife and its three precursors (between 3.9 Ma and ~12 Ma, fusion of the paleo‐islands ~3.5 Ma) and has a less complex geological history (Carracedo et al. 2002). However, equal to Tenerife, La Palma exhibits a high potential for the formation of reproductive isolation between plant populations, due to spatial (topographical heterogeneity, but also Euclidian distance) and ecological factors (strong gradients, e.g., elevation/temperature, precipitation). This makes La Palma an ideal system to investigate population genetic patterns and their relation to geographical and ecological landscape factors within species.

Species that cope with a broad range of environmental settings, *that is,* habitat generalist species, are expected to feature more influence of divergent selection among populations compared to specialized species with populations experiencing less diverse environments (Groot et al. 2011). Literature also suggests that the effects of isolated habitats and putatively lower effective population sizes in specialist species lead to higher neutral genetic differentiation among their populations (e.g., Groot et al. 2011; Gil‐López et al. 2014; Li et al. 2014). However, this oversimplifies population features of specialist versus generalist species and has rarely been directly evaluated for plant species (but see Gil‐López et al. 2014). To address the role of ecological amplitudes for genetic structures within *Aeonium* taxa and the potential of within‐island evolutionary divergence on La Palma, we investigated range‐wide population genetic structures of a wide‐spread generalist species (*Ae. davidbramwellii*) and a spatially and ecologically more specialized species (*Ae. nobile*).

The following two hypotheses were tested:

- The topographical heterogeneity and the related spatial and ecological isolation of *Aeonium* populations are reflected in intraspecific genetic structuring.
- The generalist and wide‐spread species (*Ae. davidbramwellii*) shows a higher genetic population differentiation compared to the specialist species (*Ae. nobile*).

## Materials and Methods

### Study area

La Palma (706 km^2^, between 28°27′–28°51′ N and 17°43′–18°0′ W) is the northwesternmost and second youngest island of the Canarian archipelago (Carracedo et al. 2002). The northern part of La Palma is dominated by an extinct shield volcano (2426 m a.s.l. on the highest peak) with a large central erosional depression (Caldera de Taburiente) that opens to the southwest (Barranco de las Angustias) and a complex radial network of deep erosion valleys (Barrancos) dissecting its outer flanks (see Fig. 2B). The southern part is geologically younger with a volcanic ridge system starting from the Caldera de Taburiente and running out to the southern tip of the island, where active volcanism still occurs (Carracedo et al. 2002). On the western flanks south of the Caldera, the past Cumbre Nueva mega‐landslide (~560 ka) created a comparably gently sloping landscape (Carracedo et al. 1999; Colmenero et al. 2012).

La Palma shows a Mediterranean subtropical climate of dry summer and more rainy winter seasons. Nevertheless, the high topographical structures generate distinct rain shadow effects with strong differences between the humid northeastern island sections and dry southwestern parts.

### Study species and sampling design

The genus *Aeonium* webb & berthel. consists of leaf‐succulent long‐lived perennial herbs or small shrubs with a great diversity of growth forms, ecological niches, and physiological attributes (Lems 1960; Liu 1989; Lösch 1990). Large inflorescences with numerous colored and nectariferous flowers imply entomophily and outcrossing (Esfeld et al. 2009). Additionally, *Aeonium* species produce relative small (0.4–0.6 mm long) and light (0.02–0.04 mg) seeds, suggesting some suitability for wind dispersal (Liu 1989; Vazačová and Münzbergová 2014). Pollination by insects and wind dispersal of seeds might facilitate common exchange of alleles and genotypes between nearby populations. However, specialized seed traits that would advance anemochory or other potential long‐distance dispersal are lacking, and dispersal capabilities of *Aeonium* species have been shown only moderate (Vazačová and Münzbergová 2014). Thus, gravity is probably the most important dispersal agent besides more or less occasional wind drift events.

**Figure 1:**
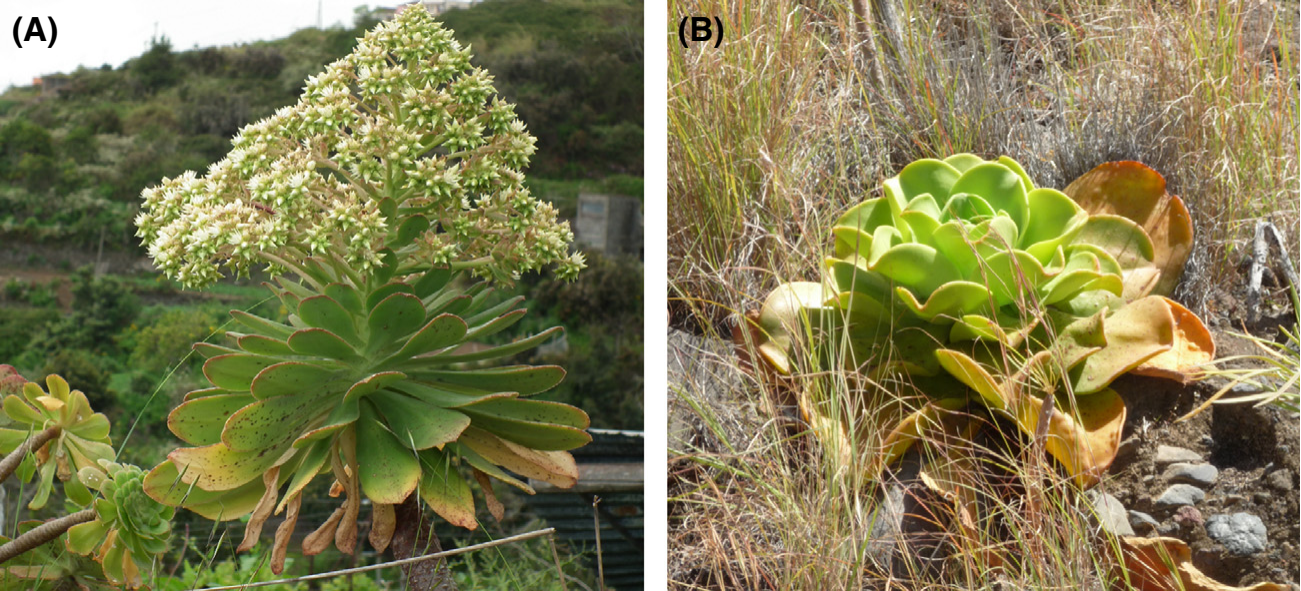
The two study species *Aeonium davidbramwellii* (A) and *Aeonium nobile* (B). Photographs by Carl Beierkuhnlein and Katharina Staab, respectively.

**Figure 2:**
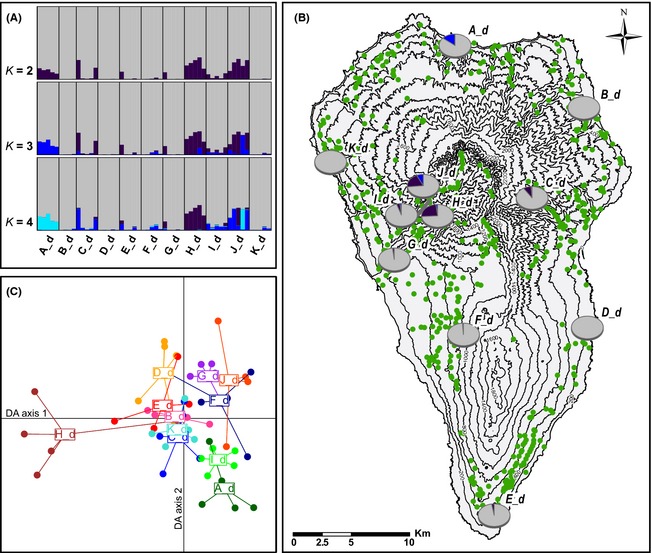
Genetic clustering results for *Aeonium davidbramwellii*. (A) structure results for *K* = 2–4. Vertical bars depict color‐coded proportions of genetic cluster assignments for single individuals with populations being separated by vertical black lines. (B) Map of La Palma showing the occurrences of the species (green points) and the genetic structuring from the structure results for *K* = 3 as pie charts per analyzed population. (C) Discriminant analysis of principal components ordination plot with each point representing one individual, distributed along the two first linear Discriminants. Individuals of the same population are connected to its centroid and share the same coloring. Five principal components were retained (representing 31.6% of the total variation) to obtain adequate discrimination of clusters, based on cross‐validation. Populations are named by their clockwise position around the island, starting from the north.

*Aeonium davidbramwellii* H.‐Y. liu (Fig. 1A) is a generalist species, growing on rocks, soil banks, and cliffs throughout almost every part of La Palma from 0 to 1000 m a.s.l. (Liu 1989) and up to 1800 m a.s.l. (own observations, see also Fig. 2B). It forms distinct populations but also occurs as scattered individuals and seems to be adapted to a wide range of environmental situations on the island. *Ae. davidbramwellii* is a subshrub, mostly with a polycarpic life cycle, and is reported diploid with 2*n* = 36 (Liu 1989).

*Aeonium nobile* (praeger) praeger (Fig. 1B) realizes a far more narrow ecological niche, due to its growth site restrictions to dry slopes, banks, and cliffs with high insolation (Liu 1989). It is thus much rarer than *Ae. davidbramwellii* and occurs in distinct and mostly small populations from 0 to 750 m a.s.l. (Liu 1989) and up to 1200 m a.s.l. (own observations). The current range is largely limited to the western sectors of La Palma showing a disjunction into a northern and a southern distribution, separated by the landslide area (see Fig. 4B). However, one population is present in the east of La Palma, possibly representing the remnant of a larger occurrence area in the past (see Voggenreiter 1974; Liu 1989). *Ae. nobile* individuals are monocarpic and show only one large succulent leaf rosette and have a diploid chromosome set of 2*n* = 36 (Liu 1989).

There is no indication for a sister taxon relationship between the two study species. According to a recent analysis of ITS data (Kondraskov et al. 2015), they are separately placed within a clade including six additional species with distinct distributions on four islands of the Canaries. The interspecific relationships are, however, poorly supported. In contrast, Liu's (1989) morphological analyses showed *Ae. nobile* as a rather distant relative of *Ae. davidbramwellii*, more related to *Aeonium* species distributed outside the Canary Islands. It is thus unlikely that the two species derived from each other, but rather that they derived from different ancestors subsequent to independent colonization events of La Palma.

**Figure 3:**
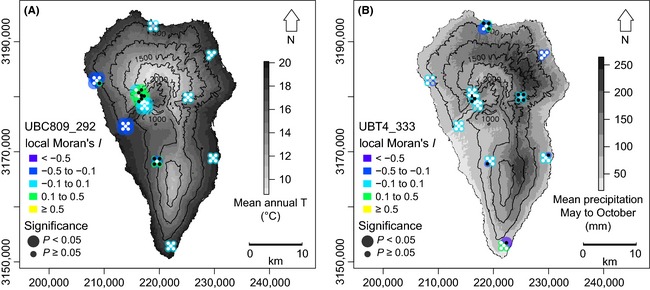
Potential adaptive genetic differentiation among populations of *Aeonium davidbramwellii* of locus UBC809_292 in relation to mean annual temperature (A) and of locus UBT4_333 in relation to mean precipitation from May to October (B) on La Palma. Black, white, and gray dots indicate the presence or absence of the respective Inter‐Simple Sequence Repeat fragment in an individual or missing data, respectively. Local Moran's *I* values >0.1 indicate local spatial autocorrelation of allelic variation. Single individual coordinates were dispersed around their population center for mapping purposes.

The sampling aimed to cover the entire distribution of the two species, including potential effects of topographical gene flow barriers and environmental variation within the ranges, respectively. In each population, five distantly growing individuals were chosen arbitrarily to avoid sampling of close relatives and to cover the whole populations' variability. Leaf samples were dried and stored in silica gel. This resulted in 11 populations and 55 individuals sampled for *Ae. davidbramwellii* and ten populations with 50 individuals in total sampled for *Ae. nobile*. The DNA sampling was accompanied by a species mapping project (see Irl et al. in press for details), revealing 597 and 70 occurrence points for *Ae. davidbramwellii* and *Ae. nobile* on the entire island, respectively (see Figs. 1B, 3B).

### DNA extraction and genotyping

Genomic DNA was extracted from leaf tissue using the NucleoMag 96 Plant Kit (Macherey‐Nagel, Düren, Germany), adapted to the Fastprep tissue homogenizer (FP120, MP Biomedicals Europe, Illkirch, France) and the Kingfisher magnetic particle processor (Thermo Scientific, Langenselbold, Germany): Leaf tissues (ca. 200 mg) were homogenized in 200 *μ*L buffer MC1 for 40 sec at a speed set of 6 m/sec; additional 300 *μ*L Buffer MC1 and 10 *μ*L RNase A were added to the viscous homogenate and incubated at 56°C for 30 min. Insoluble tissue debris was pelleted by centrifugation for 5 min at room temperature, and clear supernatant was used to purify genomic DNA as described in Table S1.

We applied ISSR (Inter‐Simple Sequence Repeat) markers with anchored primers to generate genetic fingerprints, a method that has been proven useful for population genetic analyses of nonmodel species due its capacity to generate highly polymorphic data with high reproducibility and cost efficiency (Zietkiewicz et al. 1994; Nybom 2004).

In a prestudy, 30 primers were screened in subsets of 20 samples of each study species, respectively, for their usefulness regarding a clear polymorphic and reliable band pattern generation on ethidium bromide‐stained agarose gels (see Table S2 for primer sequences, annealing temperatures, and references). If available, annealing temperatures were taken from the literature; otherwise, gradient PCRs (45–60°C annealing temperatures) were conducted and fingerprint patterns were compared to infer the best conditions for the primers, respectively. The same selection of nine markers turned out to be optimal for both species for the main study (see Table S2).

Selected ISSR markers (see Table S2) were amplified from genomic DNA in a 12.5 *μ*L reaction volume using the KAPA3G Plant PCR Kit (KAPA BIOSYSTEMS, Wilmington, MA): each reaction contained 1× KAPA Plant PCR buffer, 0.5× KAPA Plant PCR Enhancer, 0.3 *μ*mol/L fluorescently labeled ISSR primer (see Table S2), and 0.25 U KAPA3G Plant DNA Polymerase. The PCR profile consisted of an initial denaturation step at 95°C for 3 min, followed by 40 PCR cycles (95°C for 30 sec, primer‐specific annealing temperatures for 30 sec, 72°C for 30 sec), and a final extension step at 72°C for 5 min. Amplified ISSR fragments were mixed with the MapMarker size standard (50–1200 bp, Bioventures Inc., Murfreesboro, TN) and separated on a capillary electrophoresis system (GenomeLab GeXP Genetic Analysis System; AB Sciex Germany GmbH, Darmstadt, Germany) using a protocol for long DNA fragments as recommended by the manufacturer.

Electropherograms were processed and analyzed with genemarker 1.95 (SoftGenetics, State College, PA). The suggested binning was checked and carefully corrected by hand for each preliminary locus, before exporting the peak height tables. These were again processed manually, by specifying thresholds for minimum peak heights for each locus, based on the frequency distributions of its peak heights, respectively, as well as on thresholds for minimum peak number and minimum mean peak height for individual samples. Loci and samples that did not reach the respective thresholds, as well as monomorphic and uninformative loci, were stringently discarded, resulting in very conservative assignments of presences/absences of single ISSR fragments for the final binary matrices of our two study species, respectively.

### Data analyses

The two species datasets were analyzed equally. AFLP‐SURV (Vekemans 2002; Vekemans et al. 2002) was used to calculate overall genetic diversity *H*_t_, as well as percentage of polymorphic loci *PLP* and Nei's gene diversity *H*_e_ within populations. Additionally, allelic diversity *A* and number of alleles unique to a population (private alleles) were calculated with genalex 6.5 (Peakall and Smouse 2012). This program was also used to infer overall population differentiation, applying the *Φ*_ST_ statistics with 9999 permutations for significance testing. Furthermore, average frequency‐down‐weighted marker values (*DW*; see Schönswetter and Tribsch 2005) were calculated using AFLPdat (Ehrich 2006) to measure the amount of rare alleles within populations.

To analyze population structures, we used two nonhierarchical genetic clustering methods. First, the Bayesian algorithm implemented in structure 2.3.4 (Pritchard et al. 2000; Falush et al. 2007) was applied to infer gene pool differentiation and admixture of gene pools within individuals. Numbers of possible gene pools (*K*) ranging from 1 to 11 for *Ae. davidbramwellii* and from 1 to 10 for *Ae. nobile* were tested under the admixture setting. We used the independent allele frequency model to avoid overestimation of gene pool differentiation, but allowed for inclusion of the population origin as prior information in the models (LOCPRIOR; Hubisz et al. 2009). For each *K*, 20 runs were performed with 100,000 generations after a burn‐in period of 50,000 runs. The outputs were processed and analyzed using structure harvester (Earl and vonHoldt 2012) implementing the method of Evanno et al. (2005). The results were averaged for a particular *K* using clumpp (Jakobsson and Rosenberg 2007) and visualized using distruct (Rosenberg 2004). Geographical display of structure results was performed with arcinfo 10.0 (ESRI Inc., Redlands, CA). Second, DAPC (discriminant analysis of principal components; Jombart et al. 2010) were carried out using adegenet 1.4‐2 (Jombart et al. 2014) with population assignments as grouping factor within each species to reveal the genetic relationships among and within populations. Implemented cross‐validations were applied to choose the number of principal components in order to obtain the necessary amount of genetic variation and at the same time prevent overfitting of the discriminant functions.

To test for IBD (isolation‐by‐distance) patterns, pairwise differentiation indices (*F*_ST_) were calculated with 10,000 permutations in AFLP‐SURV, using the Bayesian method with nonuniform prior allele frequency distribution and assuming Hardy–Weinberg equilibrium (*F*_IS_ = 0, due to supposed outcrossing in the two study species). Linearized *F*_ST_ values were then correlated with logarithmized (log_10_) pairwise geographical distances (Rousset 1997) in a Mantel test, performed in genalex with 9999 permutations.

Further on, we screened the genetic variation in *Ae. davidbramwellii* and *Ae. nobile* for signatures of divergent selection due to the environmental heterogeneity on La Palma. For this purpose, we applied a combination of two different outlier loci detection methods and two correlative approaches to test potential loci–environment associations:

bayescan (Foll and Gaggiotti 2008) uses a Bayesian framework to estimate *F*_ST_ coefficients and decompose them into a population‐specific component, shared by all loci, and a locus‐specific component, shared by all populations. Then for every locus, a selection model versus a neutrality model is compared, checking whether locus‐specific components are necessary to explain the *F*_ST_. Analyses were performed with 10*50,000 iterations after a burn‐in of 100,000 iterations and twenty pilot runs with 10,000 iterations to infer proposal parameter distributions, respectively. Prior odds for the neutral model were set to 1, assuming an equal likelihood for loci to be under selection versus being not under selection, and a false discovery rate of 0.15 was used for results processing. mcheza (Antao and Beaumont 2011) takes use of the interrelationship of heterozygosity and *F*_ST_ (deficiency of *H*_e_). The software generates a null sampling distribution of *F*_ST_ estimates based on neutral expectations and then compares these simulated data with the sampled data. We employed 100,000 iterations for our analyses, using the two recommended algorithm extensions “Force mean *F*_ST_” and “Neutral mean *F*_ST_.”

We tested the full sets of loci for possible associations to eight environmental variables, which we assumed to state strong and putatively evolutionary relevant ecological gradients among the populations of our two study species (see headline of Table 2 for tested variables). MAT (Mean annual temperature) and mean annual precipitation were interpolated using data collected from 214 and 288 meteorological stations for the Canary Islands, respectively (see Irl et al. in press for sources and processing of raw data). Linear regression kriging (R package gstat; Pebesma and Graeler 2014) was applied to interpolate the variables with a spatial resolution of 100 m × 100 m, with elevation, slope, island, micro‐, and macro‐aspect as co‐variables, obtained from a DEM (digital elevation model, resolution 2 m × 2 m). Monthly precipitation data came from 47 meteorological stations (time span: 1969–1998) and were interpolated using the same linear regression kriging technique. Rainfall seasonality was computed following the index of Walsh and Lawler (1981). Annual solar radiation (Wh/m^2^/a) was calculated with the Area Solar Radiation tool in arcinfo 10.0, based on the 2 m × 2 m DEM, and values were extracted as area averages of 25 m radius circles around population coordinates, respectively.

Loci–environment association tests with LFMM (Latent Factor Mixed Modelling; Frichot et al. 2013) use a hierarchical Bayesian mixed model based on a variant of principal component analysis in which residual population structure is introduced via unobserved or latent factors. Analyses were carried out with 10 runs per environmental variable, each with 100,000 iterations, including a burn‐in of 50,000 iterations. According to the found dominance of one cluster in all individuals in the previous structure analyses, we decided to set only one latent factor (one gene pool) to correct for background population structure in the LFMM analyses. Of the 10 runs, the run with the lowest Deviance Information Criterion was selected for each variable, respectively. sam*β*ada tests logistic regression models to identify possible loci–environment associations (Stucki et al. 2014). We ran simple univariate models for each single environmental variable, respectively. Significance of model outputs was assessed taking the implemented log‐likelihood ratios (*G*‐scores) into account and was provided as *P*‐values of their *Χ* ^2^‐tests (see also Joost et al. 2007).

For loci showing consistent signatures of divergent selection (i.e., detection by several methods), the distribution of their allelic variants was geographically displayed in relation to the associated environmental variable suggested by the correlative methods. Additionally, the spatial clustering of their allelic variants was quantified by overall spatial autocorrelation (Moran's *I*) and univariate Local Indicators of Spatial Association (i.e., local Moran's *I*; Anselin 1995), both implemented in sam*β*ada. We used a weighting scheme based on a Gaussian kernel with 10 km distance for the neighbor comparisons to account for the small scales of our study area and the used sampling design. Creation of LISA maps as well as DAPC analyses was performed in R 3.1.0 (R Development Core Team 2014).

## Results

### *Aeonium davidbramwellii*

The ISSR genotyping for *Ae. davidbramwellii* resulted in 54 individuals successfully scored for 232 loci. Overall genetic diversity was low (*H*_t_ = 0.116), and population differentiation was moderate with *Φ*_ST_ = 0.104 (*P *<* *0.0001).

The Bayesian structure analysis revealed that *K* = 3 was the most likely number of genetic clusters within *Ae. davidbramwellii*, although *K* = 2 and *K* = 4 also showed high likelihoods, and Evanno's Δ*K* had its highest value at *K* = 2 (Fig. S1). Genetic structure was dominated by one cluster which determined major parts of the genotypes of all individuals (Fig. 2A, B). However, populations H_d and J_d in the Barranco de las Angustias and its northern edge, as well as C_d in the Barranco de la Madera in the west and A_d in the Barranco Fagundo in the north of La Palma, showed considerable assignment proportions to further gene pools. Considering *K* = 3, A_d was differentiated from other populations by having large portions of a cluster that furthermore only contributed to the genetic makeup of population J_d. The same was true for *K = 4*, which also showed a differentiation of H_d (Fig. 2A).

The differentiation of H_d, A_d, and J_d was also evident in the DAPC, showing these populations in different outer regions of the ordination plot (Fig. 2C), which reflects the results of the structure analysis for *K* = 3 and *K* = 4. However, A_d was more related to I_d than to J_d, which grouped loosely with G_d and I_d which were rather nondifferentiated in structure.

We found no clear IBD pattern among populations of *Ae. davidbramwellii* on La Palma: The correlation of pairwise spatial distances and pairwise genetic differentiation revealed no significant correlation (Mantel's *R* = 0.270, *P* = 0.082; Fig. S2).

Highest genetic diversity values for *A*,* PLP,* and *H*_e_ were found in populations J_d and H_d, both located within or near the Caldera de Taburiente and Barranco de las Angustias, followed by the two differentiated populations outside this region, A_d and C_d, and by I_d, also from the Barranco de las Angustias (Table 1). We found comparatively high numbers of private alleles in this region, with seven alleles unique to H_d, the highest value among all populations, and two alleles unique to G_d and I_d, respectively. However, J_d had no private allele at all, and A_d from the northern sector of La Palma had six private alleles. In contrast to the lacking private alleles, J_d had the highest value of overall rarity of alleles (*DW*), followed by H_d, A_d, C_d, and I_d. G_d from the lower Barranco de las Angustias revealed low amounts of rare alleles (Table 1).

**Table 1:** Descriptive population genetic parameters for the analyzed populations of *Aeonium davidbramwellii*

Population	Longitude UTM	Latitude UTM	Elevation (m a.s.l.)	*N*	No. of bands	No. of private alleles	*DW*	*A*	*PLP*	*H*_e_
A_d	218814	3192964	80	5	89	6	5.985	0.746	38.4	0.137
B_d	229864	3187626	134	4	51	0	2.661	0.435	22	0.075
C_d	225423	3179899	870	5	88	1	4.878	0.737	37.9	0.134
D_d	230166	3168798	85	5	58	0	2.750	0.474	25	0.087
E_d	222168	3152717	235	5	68	1	3.209	0.582	29.3	0.083
F_d	219540	3168200	893	5	66	0	3.014	0.565	28.4	0.098
G_d	213600	3174629	115	5	61	2	2.881	0.504	26.3	0.091
H_d	217192	3178367	365	5	106	7	7.181	0.905	45.7	0.165
I_d	216611	3179654	1051	5	77	2	4.166	0.642	33.2	0.104
J_d	216095	3180765	1922	5	124	0	7.543	1.047	53.4	0.185
K_d	208142	3182987	271	5	54	1	2.664	0.444	23.3	0.082

N, Number of scored individuals; *DW*, rarity index; *A*, allelic diversity; *PLP*, percentage of polymorphic loci; *H*_e_, Nei's gene diversity.

The scan for non‐neutral genetic variation resulted in indications for divergent selection on at least two loci, although there was inconsistency among the results. bayescan found three candidate loci (Table 2, green‐colored cells), which were also suggested by mcheza, additional to eight further candidates (Table 2, blue‐colored cells). The correlative methods implemented in LFMM and sam*β*ada found the most significant (*P *<* *0.001) indication of environmental dependence for loci UBC809_292 (MAT, mean precipitation seasonality) and UBT4_333 (mean precipitation from June to August and from May to October). However, while UBC809_292 was also suggested as outlier by both bayescan (*PP* = 0.79) and mcheza (*F*_ST_ = 0.25, *P *=* *0.96), UBT4_333 was detected only by mcheza (*F*_ST_ = 0.32, *P* = 0.99).

**Table 2:**
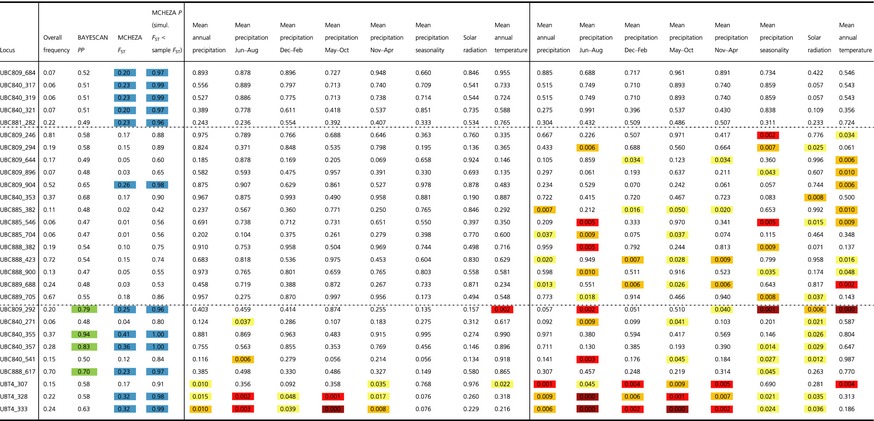
Combined results of outlier loci detection methods for *Aeonium davidbramwellii*. Locus names and overall frequencies of dominant fragments are depicted in the first (left) panel. The second panel depicts results of the *F*_ST_‐based methods (only indications for divergent selection shown): bayescan (posterior probabilities, *PP*) and mcheza (*F*_ST_‐ and *P*‐values). The third and fourth panels show results of the two correlative methods: LFMM (for K = 1) and sam*β*ada, respectively. Outlier loci candidates are highlighted by coloring in the respective columns/panels. Significance of correlations between allelic variation and environmental variables by LFMM and sam*β*ada is color‐coded as follows: yellow: *P *<* *0.05, orange: *P *<* *0.01, red: *P *<* *0.005, dark red: *P *<* *0.001

Neither allelic variants in UBC809_292 nor those in UBT4_333 showed significant overall spatial autocorrelation (Moran's *I* = 0.007, *P* = 0.110 and Moran's *I* = −0.045, *P* = 0.440, respectively). However, a clustered pattern of the dominant allele of UBC809_292 in high elevations (i.e., low temperature regions) on the western side of La Palma was obvious (Fig. 3A). Nevertheless, the segregation was not entirely consistent, resulting in only partially significant and moderately positive local spatial autocorrelation (local Moran's *I* from 0.1 to 0.5; see Fig. 3A). In UBT4_333, the allelic distribution was broader, covering populations of nearly all island regions (Fig. 3B). The dominant allele showed major contributions to populations in regions of high summer precipitation, but single individuals in populations in regions of medium precipitation, however, also bore this allele, resulting in a mixed spatial pattern and largely missing spatial autocorrelation.

### *Aeonium nobile*

For *Ae. nobile*, 44 individuals were successfully scored for 196 ISSR loci. Overall genetic diversity was on a similarly low level as for the former species (*H*_t_ = 0.115), and overall population differentiation was a bit lower (*Φ*_ST_ = 0.092, *P *<* *0.0001).

**Figure 4:**
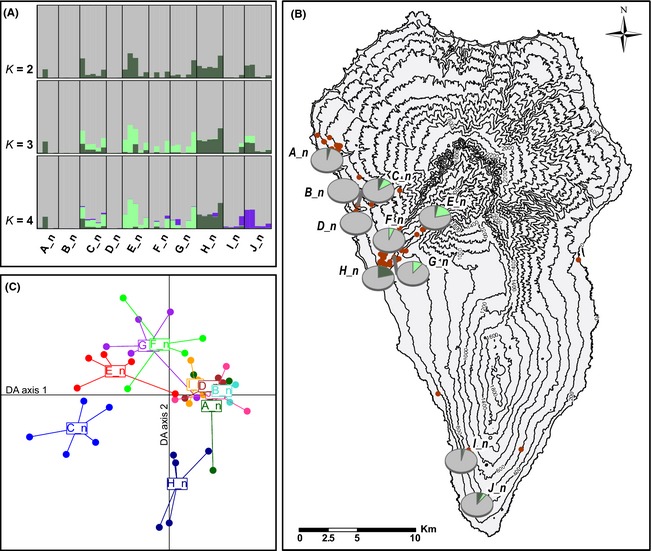
Genetic clustering results for *Aeonium nobile*. (A) structure results for *K* = 2–4. Vertical bars depict color‐coded proportions of genetic cluster assignments for single individuals with populations being separated by vertical black lines. (B) Map of La Palma showing the occurrences of the species (brown points) and the genetic structuring from the structure results for *K* = 3 as pie charts per analyzed population. (C) Discriminant analysis of principal components ordination plot with each point representing one individual, distributed along the two first linear discriminants. Individuals of the same population are connected to its centroid and share the same coloring. Ten principal components were retained (representing 37.7% of the total variation) to obtain adequate discrimination of clusters, based on cross‐validation. Populations are named by their latitudinal position, starting from the north.

The clustering analyses in structure resulted in *K* = 3 as the most likely subdividing genetic structure in *Ae. nobile*, with *K* = 2 and *K* = 4 showed high likelihoods as well and Δ*K* peaking at *K* = 2 (Fig. S3). All populations and individuals were dominated by one genetic cluster (Fig. 4A). However, populations in the Barranco de las Angustias and its northern edge (E_n, G_n, H_n, and F_n), as well as the southern populations (J_n and I_n) and C_n on the western slope of the Taburiente volcano edifice, showed some proportions of a second and a third cluster. In particular, population H_n in the lowest part of the Barranco de las Angustias showed strong proportions of the second cluster consistently among its individuals. With *K* = 4, the southernmost J_n and I_n were differentiated from the other populations, and E_n in the upper part of the Barranco de las Angustias became more differentiated in the results (Fig. 4 A, B).

The DAPC supported the differentiated state of H_n and put the other populations of the Barranco de las Angustias together in a separate group, too (Fig. 4C). However, J_n and I_n were not differentiated from the northern populations in this analyses and C_n took a very differentiated position as well.

The correlation between pairwise geographical distances and pairwise genetic differentiation between populations revealed the absence of an IBD pattern (Mantel's *R* = −0.071, *P* = 0.397, see Fig. S4).

We found the highest genetic diversity values (*A*,* PLP* and *H*_e_) in H_n, followed by the two other populations from within the Barranco de las Angustias (E_n and G_n) and by populations C_n (Table 3). F_n and the southern range populations J_n and I_n showed intermediate diversity values, while the northern populations D_n, B_n, and A_n were least diverse (with D_n consisting of only three genotyped individuals, however). H_n had five private alleles, whereas E_n and G_n had no population‐specific alleles. The second highest number of three unique alleles was found in the southernmost J_n, and C_n as well as the northernmost A_n had two private alleles. According to *DW*, all populations from the Barranco de las Angustias region showed high values, with H_n and E_n the highest. Other high values were obvious in C_n, J_n, and I_n.

**Table 3:** Descriptive population genetic parameters for the analyzed populations of *Aeonium nobile*

Population	Longitude UTM	Latitude UTM	Elevation (m a.s.l.)	N	No. of bands	No. of private alleles	*DW*	*A*	*PLP*	*H*_e_
A_n	207834	3182960	36	4	50	2	3.125	0.500	25.5	0.100
B_n	209258	3180361	293	4	37	1	2.573	0.378	18.9	0.069
C_n	210473	3179117	443	5	79	2	5.202	0.791	40.3	0.143
D_n	210327	3178565	149	3	18	1	1.882	0.179	9.2	0.045
E_n	217096	3178041	363	5	96	0	6.306	0.954	49	0.162
F_n	213028	3175880	720	4	55	1	3.840	0.546	28.1	0.109
G_n	213600	3174629	115	5	80	0	4.442	0.806	40.8	0.123
H_n	212226	3172861	6	5	103	5	6.871	1.041	52.6	0.160
I_n	219344	3157172	275	4	55	1	3.965	0.556	28.1	0.099
J_n	220699	3153422	415	5	60	3	4.448	0.592	30.6	0.108

N, Number of scored individuals; *DW*, rarity index; *A*, allelic diversity; *PLP*, percentage of polymorphic loci; *H*_e_, Nei's gene diversity.

Signatures of selective forces on genetic variation in *Ae. nobile* were very weak and inconsistent (Table S3). bayescan found no outlier loci under the same settings that were applied for *Ae. davidbramwellii*. mcheza suggested 11 loci to show variation caused by divergent selection, which, however, were only poorly supported by the two correlative methods used: LFMM found only few correlations with very low significance (*P*‐values between 0.01 and 0.05), and similarly, the correlations indicated by sam*β*ada did not reach the significance levels obtained for *Ae. davidbramwellii* and were only partially congruent with the outlier suggestions of mcheza. The most significant correlation (*P* = 0.001) was found between the allelic variation in locus UBC809_562 (mcheza:* F*_ST_ = 0.23, *P* = 0.96) and mean precipitation from June to August. However, the spatial distribution of this variation was highly inconsistent and did not show a recognizable relationship to the environmental variable (not shown).

## Discussion

The project aimed to identify population genetic patterns related to geographical and ecological heterogeneity on the western Canary Island of La Palma, to make inferences on the evolutionary potential on the island scale and for potential conservational implications. Our main findings are as follows: 1. Genetic structuring within both species is low although effects of island topography and range patterns are obvious; 2. there are indications of correlations between allelic patterns and environmental heterogeneity in temperature and precipitation variables for *Ae. davidbramwellii*; 3. the differing niche widths of *Ae. davidbramwellii* versus *Ae. nobile* did not reflect in differences in the degree of genetic structuring; however, in the generalist species *Ae. davidbramwellii* signatures of selection were more distinct.

### Population structures

The weak to moderate overall genetic population structures in *Ae. davidbramwellii* and *Ae. nobile* may be due to the following (nonexclusive) reasons:

First, lineage differentiation might be impeded by extensive gene flow. However, the moderate *Φ*_ST_ values but largely missing IBD patterns speak against common gene flow between adjacent populations, but rather suggest random (including long‐distance) dispersal events within recent timescales and limited gene flow after the establishment of populations (Slatkin 1993; Hutchison and Templeton 1999). The facts that populations of the analyzed species show rather discrete distributions over a topographically highly structured landscape, very variable population sizes (pers. obs.), and only moderate dispersal capacity (Vazačová and Münzbergová 2014) support this interpretation.

Second, recent species spreads and limited lineage differentiation also correspond with a relatively low evolutionary age of the analyzed species. Available molecular phylogenies of *Aeonium* place our two study species to the end of very short branches or into unresolved polytomies (Mort et al. 2002; Kim et al. 2008; Thiv et al. 2010; Kondraskov et al. 2015), suggesting rather recent species formation without sufficient time to differentiate from their respective ancestor and/or sister species. A recent Bayesian divergence time dating based on ITS sequence data (Kondraskov et al. 2015; see also Thiv et al. 2010) found a cladogenetic origin in the late Pleistocene or even later for *Ae. davidbramwellii* (mean stem age: 0.5 Ma, 95% highest posterior densities: 0.01–1.16 Ma) and a slightly older origin for *Ae. nobile* (mean stem age: 1.19 Ma, 95% highest posterior densities: 0.36–2.17 Ma). Many other *Aeonium* species show higher divergence estimates. Geologically young oceanic islands like La Palma generally can be assumed to host more young species and lineages with lower levels of population divergence (see also Kim et al. 1999; Bottin et al. 2005; Stuessy et al. 2014). The low overall genetic diversities compared to other endemic species on older islands of the Canaries support this idea, see, *for example, Atractylis preauxiana* on Gran Canaria (*H*_t_ = 0.219) and Tenerife (*H*_t_ = 0.229) (Caujapé‐Castells et al. 2008), *Gnaphalium teydeum* on Tenerife (*H*_t_ = 0.173) (González‐Pérez et al. 2008), both studies performed with RAPD analyses; or *Solanum vespertilio* on Tenerife (*H*_t_ = 0.205) and *Solanum lidii* on Gran Canaria (*H*_t_ = 0.207) observed with AFLP data (Prohens et al. 2007).

Third, it is also possible that the low overall genetic diversity and differentiation values are due to catastrophic throwbacks of range expansions and bottleneck events in the past of *Ae. davidbramwellii* and *Ae. nobile*. La Palma has a vivid geological history with different periods of strong volcanism, as well as multiple mega‐landslides, especially on the western side of the island (Carracedo et al. 1999; Masson et al. 2002; Colmenero et al. 2012) where the evolutionary origin of our two study species is likely. Such events have been shown in other studies to effectively shape phylogeographic trajectories on the Canary Islands (see, e.g., Brown et al. 2000; Juan et al. 2000; Emerson 2003). Violent volcanic events and mega‐landslides might have caused severe reductions in population size and interruptions in range extractions after species formation of *Ae. davidbramwellii* and *Ae. nobile*. The observed low levels of population structures can thus be interpreted as representations of initial stages of ongoing differentiation processes after late species evolution and/or delayed (stochastic) spreading over the island, but this is somewhat speculative. DNA sequence‐based phylogeographical studies and estimations of demographic histories may help to make more definite statements.

Despite low overall population differentiation within *Ae. davidbramwellii* and *Ae. nobile*, compared to a majority of Canary Island endemics (see Pérez de Paz and Caujapé‐Castells 2013), some regional structuring was detectable. In populations located in the lower Caldera de Taburiente and its large erosion valley (Barranco de las Angustias), high portions of exceptional genetic clusters, considerable differentiation, as well as the highest values of genetic diversity indices were observed for both species. The sheltered situation and special topography within the large landscape depression might have provided suitable conditions for species evolution from ancestral colonizers and for population persistence. von Gaisberg and Stierstorfer (2005) stated plant speciation centers in steep and rocky regions on El Hierro, the even younger neighboring island of La Palma, suggesting strong disturbance regimes (e.g., erosion, debris fall), low competition, and high habitat diversity as reasons for increased evolutionary processes in such habitats.

In *Ae. davidbramwellii*, a large number of private and rare alleles are located in populations within the Barranco de las Angustias and Caldera de Taburiente, also suggesting a possible evolutionary origin of the species in this region. However, the exterior populations C_d and A_d, which were situated in other deep erosion valleys (Barranco de la Madera and Barranco Fagundo, respectively), showed patterns of isolated evolution as well. This indicates a general role of topography in promoting population differentiation, which has already been shown for other plant species on the island scale (e.g., Furches et al. 2009; Riley et al. 2010). Barrier effects due to landscape structures are also a likely explanation for the nonsignificant correlation between population pairwise genetic differentiation and geographical distance in the IBD test. In general, topographical complexity and its consequences on habitat isolation and environmental heterogeneity is a commonly expected driver of speciation on oceanic islands (see, e.g., Stuessy 2007; Whittaker et al. 2008).

Despite being more restricted in its distribution than *Ae. davidbramwellii*,* Ae. nobile* showed a comparable pattern of genetic differentiation and diversity. Populations in the Barranco de las Angustias were genetically most diverse. Additionally, we found the highest density of populations and largest individual numbers in this region, pointing to a species origin here as well. However, although allele rarity (*DW*) was generally high in the Barranco de las Angustias, only population H_n in the lowest part of the Barranco showed a large number of private alleles. This population additionally was differentiated in the clustering analyses. Populations in the upper parts of the Barranco (G_n and E_n) together with F_n, located slightly behind the northern ridge of the Barranco, made up an own group, pointing to an early separation from H_n. In particular, the genetically very poor populations in the north (D_n, B_n, and A_n) give rise to the assumption that the northern part of the distribution range is the result of recent colonization by *Ae. nobile*. However, the highly differentiated position and high diversity values of population C_n suggest a relatively early dispersal event, so that C_n established before other populations in its surrounding and had time to evolve. This supports the idea of rather stochastic dispersal events from the Barranco de las Angustias.

The southernmost populations' slight genetic differentiation reflects the intra‐insular range disjunction of *Ae. nobile*, which is congruent with the geomorphological features created by the Cumbre Nueva landslide. Over a large area south of the Barranco de las Angustias, comparably mild and low structured slopes impede the occurrence of *Ae. nobile*. Additionally, the prevailing agriculture, dense settlement, and infrastructure probably pose problems for this species here. However, the low differentiation of the southern populations speaks against early vicariance due to the landslide but more for colonization from the north after this event. The recent volcanic origin of southern areas of La Palma (max. 125 ka Carracedo et al. 1999; Colmenero et al. 2012) is also more in line with a dispersal scenario. Nevertheless, range disjunctions due to landslide effects may pose an important evolutionary factor for intra‐island population divergence if large enough to prevent gene flow (see also Brown et al. 2006; Mairal et al. 2015).

Unfortunately, we were not able to take samples from the eastern part of La Palma as the only identified natural population was located high on a steep cliff south of Santa Cruz de La Palma, not accessible for sampling, which, however, might have provided additional insight in the history of *Ae. nobile*.

### Adaptive variation

While many indications of non‐neutral allele distribution in our study were of low significance or were detected only by one or two of the applied approaches, some loci in *Ae. davidbramwellii* showed consistent patterns of selection effects. The strongest indications of selective effects were related to MAT and to MP (mean precipitation) during the summer months (from May to October and from June to August), variables which constitute strong environmental gradients among the analyzed populations of this species (MAT: 11.0–19.5°C; MP_May–October_: 35–196 mm; MP_June–August_: 0–16 mm).

The spatially not entirely consistent association of marker UBT4_333 to summer precipitation all over the island might indicate that its allelic variation was already present in populations during the species' spread over La Palma and that its actual differentiation potentially represents adaptive allele frequency shifts based on this standing genetic variation. The distinct succulence of *Ae. davidbramwellii* and its CAM‐based physiology (Lösch 1990; Mort et al. 2007) imply that water limitation and water‐use efficiency play crucial roles for the fitness and ecological niche of this species. Individuals in mesic conditions thus might profit from preexisting alleles that modulate water‐use traits in order to take use of increased resource availability. Selection on standing genetic variation is known as a likely mechanism of fast adaptational processes (Feder et al. 2003; Barrett and Schluter 2008). Nevertheless, the more or less island‐wide distribution of allelic variants in UBT4_333 can at most represent an early state in a potential continuum of ecological differentiation within *Ae. davidbramwellii* (Nosil 2012).

The clustering of UBC809_292 fragments in populations of low temperature habitats in high elevations only on the western side of La Palma could indicate a newly evolved adaptation. Considering the elevational niche separation among *Aeonium* species, the temperature gradient is a likely driver of evolutionary divergence within this genus (Lösch 1984; Liu 1989). Although the evolutionary potential of the found variation in UBC809_292 remains an open question, it indicates that ecological heterogeneity in combination with topography (in this case, the old Taburiente edifice and ridge systems separating eastern and western parts of the island) can foster initial population divergence on the small scale of an island. Such interactions of different habitat conditions and topographical gene flow barriers on plant population divergence on the island scale have recently been shown, *for example,* in *Elaeocarpus photiniifolia* on the Bonin Islands, Japan (Sugai et al. 2013) or in *Jumellea rossii* on Réunion, Mascarene Islands (Mallet et al. 2014).

Reciprocal ecological disadvantage of migrants and hybrids and built‐up of reproductive isolation between divergent populations is needed for further evolutionary separation (Nosil 2012; Savolainen et al. 2013). Although gene flow seems to be limited or at least highly variable among populations, genetic patterns in *Ae. davidbramwellii* yet lack signals of such reproductive isolation and show only incipient intraspecific evolutionary differentiation.

### Species differences

In the literature, specialist species are expected to have smaller and more isolated populations than related generalist species; thus, they would be more affected by genetic drift due to meta‐population dynamics, fostering higher variation among populations (e.g., Groot et al. 2011; Li et al. 2014 and references therein). Concordantly, in an allozyme data meta‐analysis for Canary Island plant endemics, Pérez de Paz and Caujapé‐Castells (2013) found significantly higher differentiation indices (*G*_ST_ and *F*_ST_) and lower genetic diversity in species occurring in small and intermediate population sizes (≤ 500 individuals) compared to species with large populations (> 500 individuals).

Contrastingly, our study revealed no significant differences in overall population structures and total genetic diversity between the generalist and the specialist species. *Ae. davidbramwellii* has a wider and more continuous distribution than *Ae. nobile* and mostly showed larger population sizes (pers. obs.). Thus, one could have expected lower population differentiation from the general model described above. Additionally, it is younger than *Ae. nobile* (Kondraskov et al. 2015); thus, its populations theoretically had less time to differentiate. However, the wide distribution of *Ae. davidbramwellii* is also characterized by a much stronger environmental heterogeneity and large‐scale topographical subdivision than that of *Ae. nobile*. We thus suppose that for the moderate population differentiation within *Ae. davidbramwellii,* the effects of a rather continuous distribution, larger population sizes, and less evolutionary age were compensated by the effects of differentiating selection pressures and strong landscape structuring. In contrast, the similar differentiation level in *Ae. nobile* might have been dominated by drift effects. In fact, although only marginally significant, a potential IBD pattern was more discernible in *Ae. davidbramwellii*, pointing to higher gene flow between neighboring populations and lower importance of drift. Also, consistent signals of differentiating selection were found only in the generalist *Ae. davidbramwellii*, supporting the hypothesized evolutionary effects of strong environmental heterogeneity within its range. In the sampled range of *Ae. nobile*, ecological gradients are limited in strength, missing divergent selection regimes among populations of this species which provides a likely explanation for the low association of allelic patterns and ecological variables. Furthermore, the only potential large‐scale landscape barrier for gene flow is the region of the ancient Cumbre Nueva landslide. This emphasizes that besides population continuity and population sizes, landscape factors and the strength of environmental heterogeneity should be considered in studies of generalist–specialist patterns, at least on small spatial scales like single islands.

It also has to be mentioned that we observed considerable regional phenotypic variation in *Ae. davidbramwellii* during our fieldwork on La Palma, which was not true for *Ae. nobile*. Especially in populations in the Caldera de Taburiente and the Barranco de las Angustias, individuals showed exceptionally strong branching, whereas in the southern regions of La Palma, branching in this species was rather uncommon. This is a further indication of divergent selection pressures, although not reflected in our genetic data.

It has been shown that plant speciation can occur by adaptive specialization of certain populations of generalist species, leading to sympatric pairs of sister species with largely different ecological niche breadths (Grossenbacher et al. 2014). This might also be an evolutionary scenario for *Ae. davidbramwellii* and some of its populations, while the specialized *Ae. nobile* actually seems to be in an “evolutionary dead end,” without great prospects of further intraspecific niche divergence and adaptive differentiation.

However, some of the drawn conclusions might have limitations due to the following two reasons: First, future studies should include more populations and a denser representation of all regions of La Palma. We included many populations from the Barranco de las Angustias region which potentially might have biased our observations of disproportionally high local genetic variation. Further on, larger sampling sizes may allow for more distinct and significant results according to genetic variation estimates within and among populations. Second, although ISSR analyses have proven to be reliable approaches for plant genotyping and population genetic studies (see, e.g., Nybom 2004) and although we put high effort in genotyping accuracy, this dominant marker system imminently has some potential for genotyping errors due to possible PCR artifacts and homoplasy. Further on, future studies would benefit from the use of haplotype sequence data which might enable a more in‐depth inference of phylogeographic and demographic histories. Genomic approaches are necessary to identify evolutionary relevant genes and their effects on phenotypic and environmental variation, as well as potential genomic mechanisms involved in population divergence. Further studies will thus help to understand the processes of adaptive evolution in this genus beyond the level of candidate markers.

Nevertheless, investigations of intraspecific variation and population structures, and therefore of recent eco‐evolutionary processes and potential drivers for species divergence in *Aeonium*, have not taken place yet. Although not definite, our study revealed basic patterns within the two analyzed species and gives additional insights into species evolution on small oceanic islands.

## Conflict of Interest

None declared.

## Supporting information


**Figure S1.** Assessment of the most likely number of clusters of structure runs using the method of Evanno et al. (2005) for *Aeonium davidbramwellii*.
**Figure S2.** Correlation of pair‐wise geographic distance and pair‐wise linearised *F*_ST_ between populations of *Aeonium davidbramwellii*: Mantel's *R* = 0.270, *P* = 0.082.
**Figure S3.** Assessment of the most likely number of clusters of structure runs using the method of Evanno et al. (2005) for *Aeonium nobile*.
**Figure S4.** Correlation of pair‐wise geographic distance and pair‐wise linearised *F*_ST_ between populations of *Aeonium nobile*: Mantel's *R* = −0.071, *P* = 0.397.

**Table S1.** Plate layout and instrument settings for DNA purification via BindIT 3.1 KingFisher software.
**Table S2.** Tested primers for the ISSR analyses, their sequences and PCR annealing temperatures.
**Table S3.** Combined results of outlier loci detection methods for *Aeonium nobile*.
