# Supplementary material for: Spatial and ecological population genetic structures within two island‐endemic Aeonium species of different niche width
Source: Ecol Evol. 2015 Sep 14;5(19):4327–44. doi: 10.1002/ece3.1682 (PMC4667834; doi:10.1002/ece3.1682)
Supplement: Supplementary file 1 — Figure S1. Assessment of the most likely number of clusters of structure runs using the method of Evanno et al. (2005) for Aeonium davidbramwellii. Figure S2. Correlation of pair‐wise geographic distance and pair‐wise linearised F ST between populations of Aeonium davidbramwellii: Mantel's R = 0.270, P = 0.082. Figure S3. Assessment of the most likely number of clusters of structure runs using the method of Evanno et al. (2005) for Aeonium nobile. Figure S4. Correlation of pair‐wise geographic distance and pair‐wise linearised F ST between populations of Aeonium nobile: Mantel's R = −0.071, P = 0.397. [file ECE3-5-4327-s001.docx]

**Appendix figures**


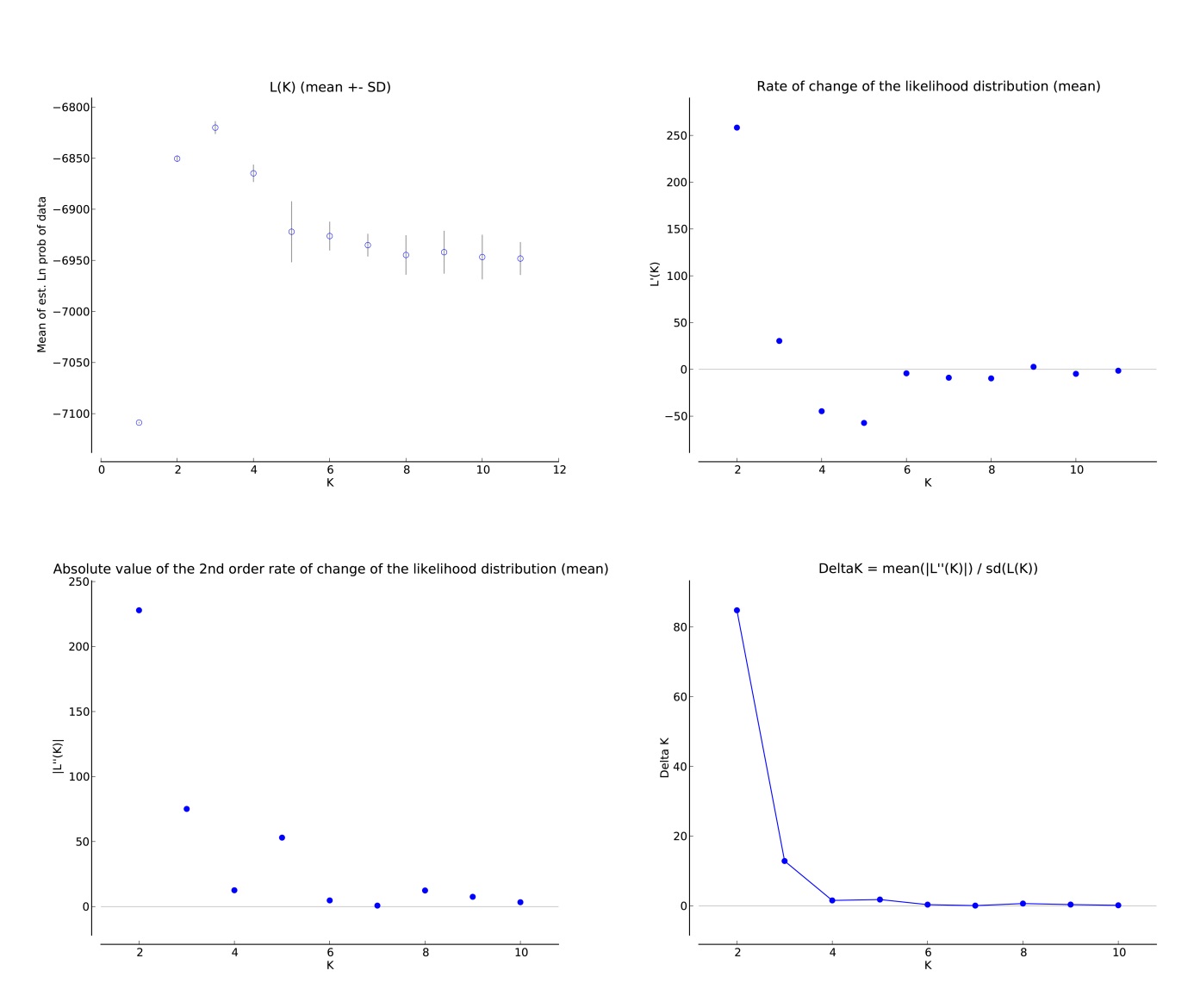


### Fig. S1: Assessment of the most likely number of clusters of Structure runs using the method of Evanno et al. (2005) for *Aeonium davidbramwellii*. Shown are means of (top left) the log probability of the data mean L(*K*), (top right) the rate of change of the likelihood distribution mean L'(*K*), (bottom left) the absolute values of the second order rate of change L''(*K*) and (bottom right) Δ*K*, over 20 runs for each *K* value, respectively. Plots created with Structure Harvester (Earl and vonHoldt 2012).


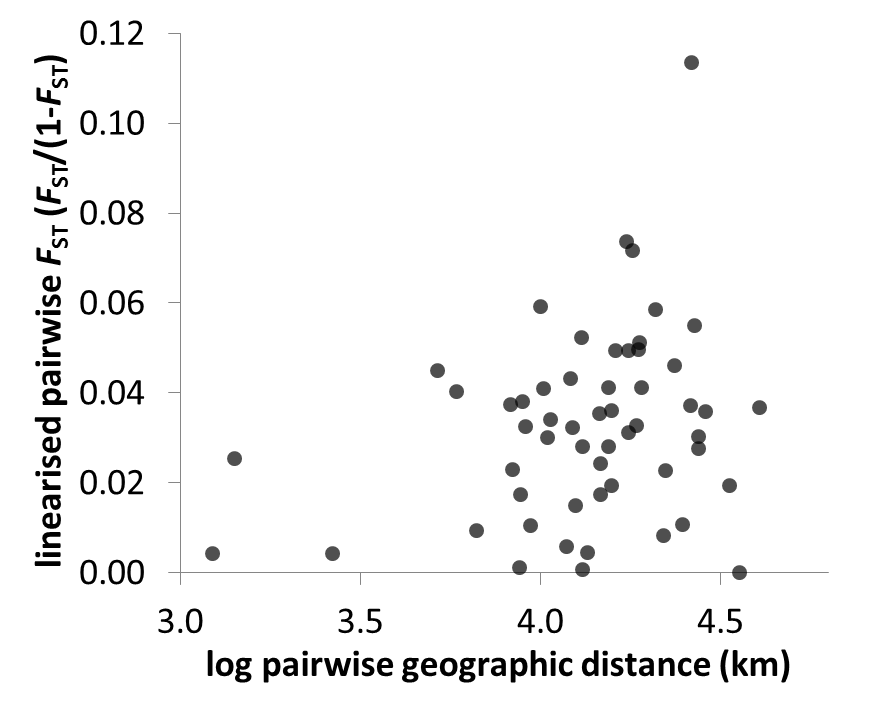


### Fig. S2: Correlation of pair-wise geographic distance and pair-wise linearised *F*_ST_ between populations of *Aeonium davidbramwellii*: Mantel’s *R* = 0.270, *P* = 0.082.


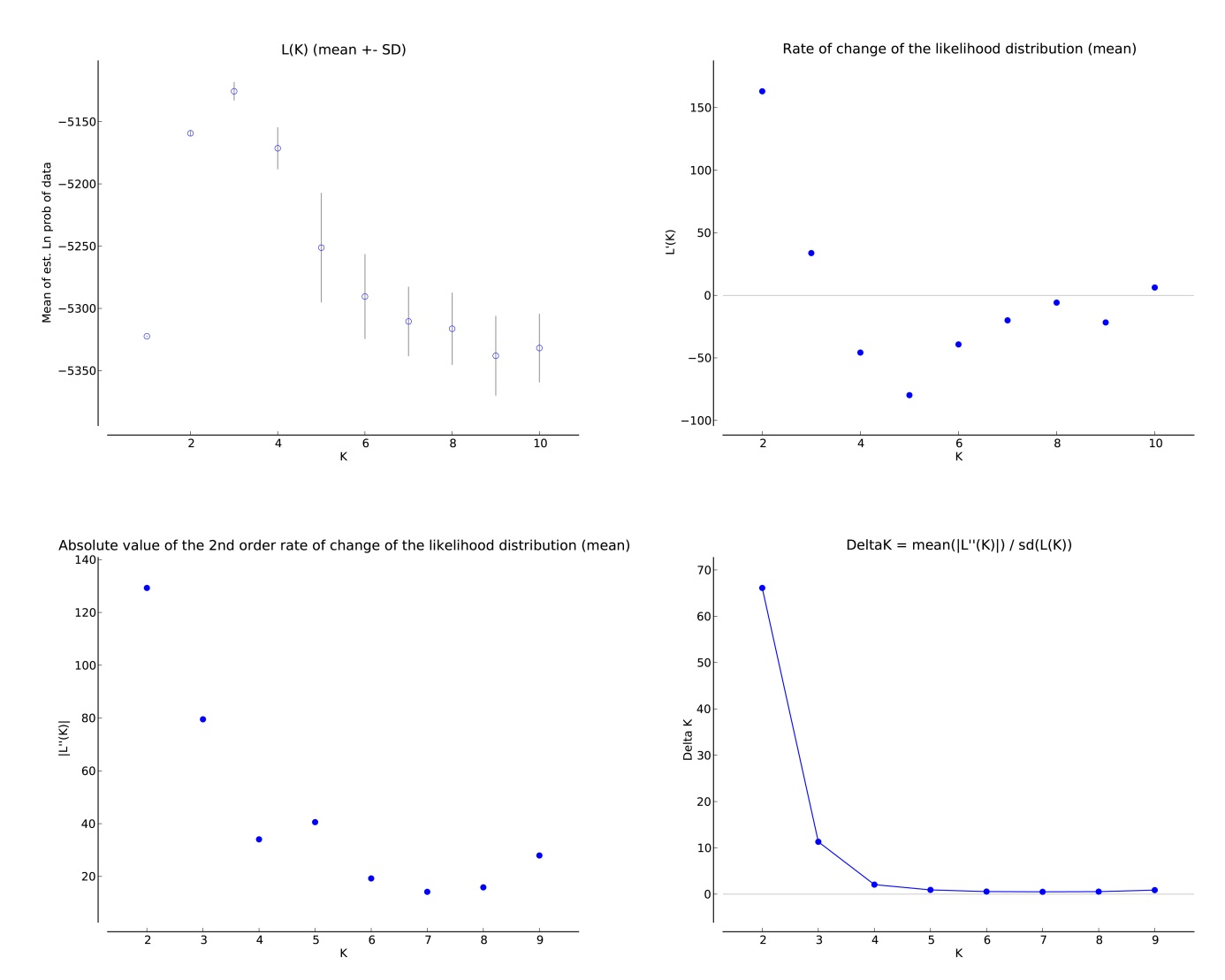


### Fig. S3: Assessment of the most likely number of clusters of Structure runs using the method of Evanno et al. (2005) for *Aeonium nobile*. Shown are means of (top left) the log probability of the data mean L(*K*), (top right) the rate of change of the likelihood distribution mean L'(*K*), (bottom left) the absolute values of the second order rate of change L''(*K*) and (bottom right) Δ*K*, over 20 runs for each *K* value, respectively. Plots created with Structure Harvester (Earl and vonHoldt 2012).


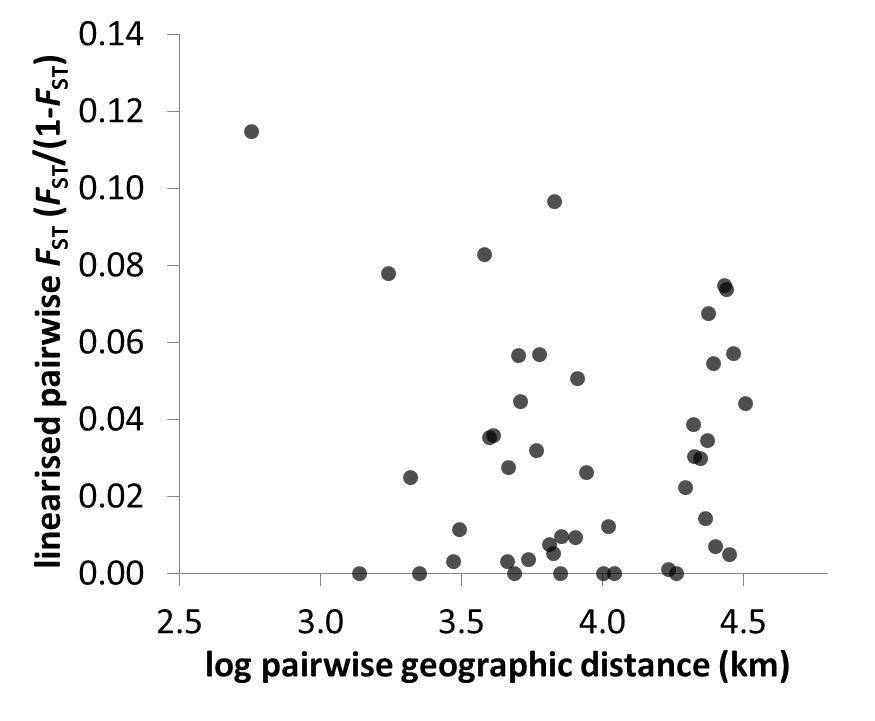


### Fig. S4: Correlation of pair-wise geographic distance and pair-wise linearised *F*_ST_ between populations of *Aeonium nobile*: Mantel’s *R* = -0.071, *P* = 0.397.
