# Supplementary material for: Spatial and ecological population genetic structures within two island‐endemic Aeonium species of different niche width
Source: Ecol Evol. 2015 Sep 14;5(19):4327–44. doi: 10.1002/ece3.1682 (PMC4667834; doi:10.1002/ece3.1682)
Supplement: Supplementary file 2 — Table S1. Plate layout and instrument settings for DNA purification via BindIT 3.1 KingFisher software. Table S2. Tested primers for the ISSR analyses, their sequences and PCR annealing temperatures. Table S3. Combined results of outlier loci detection methods for Aeonium nobile. [file ECE3-5-4327-s002.docx]

# Appendix tables

Table S1: Plate layout and instrument settings for DNA purification via BindIT 3.1 KingFisher software. After step 8, eluted DNA was transferred to plastic cups and stored at -20 °C. Due to increased homogenization volume in comparison to the original protocol, binding of genomic DNA to magnetic beads was carried out in the three initial steps. After the elution step, the magnetic beads were automatically collected and disposed.

| **Steps** |  | **Step 1**  **Bind**  **MC2** | **Step 2**  **Bind**  **MC2** | **Step 3**  **Bind**  **MC2** | **Step 4**  **Wash**  **MC3** | **Step 5**  **Wash**  **MC4** | **Step 6**  **Wash**  **Ethanol** | **Step 7**  **Wash**  **MC5** | **Step 8**  **Elution**  **MC6** |
| --- | --- | --- | --- | --- | --- | --- | --- | --- | --- |
| Layout | Row | A | B | C | D | E | F | G | H |
|  | Sample,  magnetic beads,  buffers | 92 µl supernatant  8 µl mag. beads  100 µl MC2 | 100 µl supernatant  100 µl MC2 | 100 µl supernatant  100 µl | 200 µl MC3 | 200 µl MC4 | 200 µl ethanol (80%) | 200 µl MC5 | 20 µl MC6 |
| Beginning | Precollect | no | no | no | no | no | no | no | no |
|  | Release time (mm:ss) | no release | 00:30 | 00:30 | 00:30 | 00:30 | 00:30 | no release | 00:15 |
|  | Release speed | - | fast | fast | fast | fast | fast | - | fast |
| Mixing | Pause for manual handling | no | no | no | no | no | no | no | no |
|  | Mixing time (mm:ss) | 05:00 | 05:00 | 05:00 | 01:00 | 01:00 | 01:00 | 01:00 | 10:00 |
|  | Mixing speed | medium | medium | medium | fast | fast | fast | medium | medium |
| End | Postmix | no | no | no | no | no | no | no | no |
|  | Collect count | 3 | 3 | 3 | 3 | 3 | 3 | 3 | 6 |
|  | Collect time (s) | 1.5 | 1,5 | 1,5 | 1.5 | 1.5 | 1.5 | 1.5 | 1.5 |

Table S2: Tested primers for the ISSR analyses, their sequences and PCR annealing temperatures. Used labelling (dye) for the capillary electrophoresis and are given only for the markers of the final analyses plus their fragment size spectra and numbers of polymorphic fragments obtained for the two study species, respectively. Annealing temperatures were the same for both species. Sources for primers are indicated by superscript numbers: ^1^ UBC set 9 (Biotechnology Laboratory, The University of British Columbia, Canada), ^2^ newly designed for this study, and ^3^ Wolfe *et al.* 1998, see methods. n.r.: no results.

|  |  |  |  | ***Aeonium davidbramwellii*** | | ***Aeonium nobile*** | |
| --- | --- | --- | --- | --- | --- | --- | --- |
| **Primer** | **Primer sequence** | **Fluorescent label for capillary electrophoresis** | **Annealing temperature (° C)** | **Fragment size range (bp)** | **No. polymorphic fragments** | **Fragment size range (bp)** | **No. polymorphic fragments** |
| UBC807 ^1^ | (AG)_8_-T | BMN-6 | 51 | 282-703 | 15 | 234-863 | 18 |
| UBC809 ^1^ | (AG)_8_-G | BMN-5 | 51 | 56-930 | 49 | 280-982 | 25 |
| UBC840 ^1^ | (GA)_8_-YT | BMN-5 | 48 | 271-909 | 54 | 115-1060 | 47 |
| UBC848 ^1^ | (CA)_6_-RG | DY-751 | 51 | 280-928 | 11 | 215-848 | 7 |
| UBC881 ^1^ | (GGGT)_3_-G | BMN-6 | 51 | 152-941 | 9 | 85-935 | 29 |
| UBC885 ^1^ | BHB-(GA)_7_ | BMN-6 | 45 | 232-870 | 25 | 265-859 | 19 |
| UBC888 ^1^ | BDB-(CA)_7_ | BMN-5 | 51 | 249-907 | 38 | 263-897 | 30 |
| UBC889 ^1^ | DBD-(AC)_7_ | DY-751 | 51 | 443-948 | 22 | 425-748 | 5 |
| UBT4 ^2^ | (GA)_8_-YA | DY-751 | 45 | 307-792 | 9 | 271-1089 | 16 |
| UBC812 ^1^ | (GA)_8_-A | - | 45 | - | - | - | - |
| UBC822 ^1^ | (TC)_8_-A | - | 51 | - | - | - | - |
| UBC841 ^1^ | (GA)_8_-YC | - | 51 | - | - | - | - |
| UBC842 ^1^ | (GA)_8_-YG | - | 56 | - | - | - | - |
| UBC845 ^1^ | (CT)_8_-RG | - | 45 | - | - | - | - |
| UBC850 ^1^ | (GT)_8_-YC | - | 45 | - | - | - | - |
| UBC857 ^1^ | (AC)_8_-YG | - | 45 | - | - | - | - |
| UBC859 ^1^ | (TG)_8_-RC | - | 45 | - | - | - | - |
| UBC861 ^1^ | (ACC)_6_ | - | 58 | - | - | - | - |
| UBC890 ^1^ | VHV-(GT)_7_ | - | 56 | - | - | - | - |
| UBC891 ^1^ | HVH-(TG)_7_ | - | 51 | - | - | - | - |
| UBT1 ^2^ | (AC)_8_-CH | - | 45 | - | - | - | - |
| UBT2 ^2^ | (AG)_8_-CR | - | 51 | - | - | - | - |
| UBT3 ^2^ | (CG)_8_-RC | - | n.r. | - | - | - | - |
| UBT5 ^2^ | (GC)_8_-YG | - | n.r. | - | - | - | - |
| UBT6 ^2^ | (GT)_6_-AG | - | 45 | - | - | - | - |
| UBT7 ^2^ | (GT)_8_-YT | - | 45 | - | - | - | - |
| UBT8 ^2^ | (TG)_8_-CR | - | 45 | - | - | - | - |
| UBT9 ^2^ | (TG)_8_-RG | - | 45 | - | - | - | - |
| 17901 ^3^ | (GT)_6_-YR | - | 45 | - | - | - | - |
| 17902 ^3^ | (GT)_6_-AY | - | 45 | - | - | - | - |

Code for degenerated 5’- or 3’-anchor bases: Y = C or T, R = A or G, B = C, G or T, D = A, G or T, H = A, C or T, and V = A, C or G.

**Reference**

Wolfe, Andrea D.; Xiang, Qiu-Yun; Kephart, Susan R. (1998): Assessing hybridization in natural populations of *Penstemon* (Scrophulariaceae) using hypervariable intersimple sequence repeat (ISSR) bands. *Molecular Ecology* 7, pp. 1107–1125.

Table S3: Combined results of outlier loci detection methods for *Aeonium nobile*. Locus names and overall frequencies of dominant fragments are depicted in the first (left) panel. The second panel depicts results of the *F*_ST_-based methods (only indications for divergent selection shown): Bayescan (Posterior probabilities, *PP*) and Mcheza (*F*_ST_- and *P*-values). The third and fourth panel show results of the two correlative methods LFMM (for K = 1) and Samβada, respectively. Outlier loci candidates are highlighted by colouring in the respective columns/panels. Significance of correlations between allelic variation and environmental variables by LFMM and Samβada is colour-coded as follows: yellow: *P* < 0.05, orange: *P* < 0.01, red: *P* < 0.005, dark red: *P* < 0.001.

| **locus** | **overall frequency** | **Bayescan *PP*** | **Mcheza *F*_ST_** | **Mcheza *P* (simul. *F*_ST_ < sample *F*_ST_)** | **mean annual precipitation** | **mean precipitation JUN - AUG** | **mean precipitation DEC - FEB** | **mean precipitation MAY - OCT** | **mean precipitation NOV - APR** | **mean precipitation seasonality** | **solar radiation** | **mean annual temperature** | **mean annual precipitation** | **mean precipitation JUN - AUG** | **mean precipitation DEC - FEB** | **mean precipitation MAY - OCT** | **mean precipitation NOV - APR** | **mean precipitation seasonality** | **solar radiation** | **mean annual temperature** |
| --- | --- | --- | --- | --- | --- | --- | --- | --- | --- | --- | --- | --- | --- | --- | --- | --- | --- | --- | --- | --- |
| **UBC840_404** | 0.09 | 0.48 | 0.00 | 0.53 | 0.979 | 0.934 | 0.996 | 0.701 | 0.887 | 0.538 | 0.491 | 0.865 | 0.950 | 0.858 | 0.690 | 0.515 | 0.814 | 0.429 | 0.005 | 0.425 |
| **UBC840_406** | 0.16 | 0.49 | 0.04 | 0.68 | 0.584 | 0.869 | 0.796 | 0.973 | 0.849 | 0.847 | 0.336 | 0.506 | 0.121 | 0.494 | 0.548 | 0.703 | 0.405 | 0.559 | 0.008 | 0.029 |
| **UBC840_721** | 0.07 | 0.47 | 0.04 | 0.88 | 0.328 | 0.731 | 0.633 | 0.648 | 0.585 | 0.762 | 0.834 | 0.427 | 0.004 | 0.319 | 0.038 | 0.111 | 0.036 | 0.210 | 0.439 | 0.022 |
| **UBC848_848** | 0.11 | 0.50 | 0.07 | 0.85 | 0.304 | 0.622 | 0.420 | 0.375 | 0.346 | 0.629 | 0.709 | 0.267 | 0.086 | 0.753 | 0.006 | 0.009 | 0.007 | 0.331 | 0.314 | 0.051 |
| **UBC881_487** | 0.07 | 0.47 | 0.00 | 0.64 | 0.652 | 0.410 | 0.925 | 0.947 | 0.923 | 0.743 | 0.334 | 0.661 | 0.336 | 0.082 | 0.788 | 0.993 | 0.925 | 0.250 | 0.002 | 0.384 |
| **UBC881_935** | 0.14 | 0.47 | 0.00 | 0.53 | 0.832 | 0.495 | 0.607 | 0.548 | 0.596 | 0.828 | 0.132 | 0.780 | 0.761 | 0.246 | 0.322 | 0.355 | 0.415 | 0.568 | 0.007 | 0.700 |
| **UBC888_790** | 0.05 | 0.48 | 0.05 | 0.95 | 0.223 | 0.861 | 0.217 | 0.173 | 0.130 | 0.987 | 0.559 | 0.120 | 0.026 | 0.492 | 0.009 | 0.014 | 0.009 | 0.910 | 0.690 | 0.010 |
| **UBC889_594** | 0.07 | 0.47 | 0.00 | 0.51 | 0.778 | 0.891 | 0.850 | 0.587 | 0.736 | 0.411 | 0.300 | 0.941 | 0.642 | 0.953 | 0.532 | 0.456 | 0.629 | 0.283 | 0.002 | 0.769 |
| **UBC807_379** | 0.82 | 0.62 | 0.22 | 0.96 | 0.196 | 0.542 | 0.532 | 0.338 | 0.320 | 0.131 | 0.528 | 0.239 | 0.009 | 0.056 | 0.033 | 0.075 | 0.047 | 0.016 | 0.218 | 0.021 |
| **UBC807_615** | 0.11 | 0.49 | 0.16 | 0.96 | 0.995 | 0.195 | 0.862 | 0.748 | 0.664 | 0.969 | 0.565 | 0.859 | 0.780 | 0.005 | 0.319 | 0.699 | 0.381 | 0.208 | 0.025 | 0.932 |
| **UBC807_654** | 0.52 | 0.70 | 0.21 | 0.95 | 0.960 | 0.920 | 0.923 | 0.849 | 0.911 | 0.695 | 0.921 | 0.962 | 0.891 | 0.607 | 0.879 | 0.953 | 0.949 | 0.963 | 0.765 | 0.902 |
| **UBC881_467** | 0.16 | 0.60 | 0.20 | 0.97 | 0.804 | 0.823 | 0.974 | 0.829 | 0.842 | 0.522 | 0.851 | 0.710 | 0.454 | 0.212 | 0.791 | 0.923 | 0.730 | 0.428 | 0.937 | 0.391 |
| **UBC881_476** | 0.07 | 0.50 | 0.12 | 0.97 | 0.298 | 0.543 | 0.630 | 0.595 | 0.551 | 0.200 | 0.417 | 0.276 | 0.006 | 0.077 | 0.076 | 0.149 | 0.054 | NA | 0.093 | NA |
| **UBC881_581** | 0.05 | 0.48 | 0.16 | 1.00 | 0.898 | 0.810 | 0.956 | 0.808 | 0.897 | 0.692 | 0.512 | 0.901 | 0.731 | 0.397 | 0.855 | 0.709 | 0.859 | 0.764 | 0.043 | 0.878 |
| **UBC888_470** | 0.39 | 0.51 | 0.20 | 0.95 | 0.855 | 0.670 | 0.978 | 0.858 | 0.800 | 0.677 | 0.560 | 0.673 | 0.580 | 0.082 | 0.701 | 0.959 | 0.643 | 0.937 | 0.183 | 0.307 |
| **UBC807_342** | 0.09 | 0.51 | 0.20 | 0.98 | 0.118 | 0.653 | 0.110 | 0.077 | 0.049 | 0.980 | 0.276 | 0.041 | 0.007 | 0.910 | 0.004 | 0.006 | 0.004 | 0.583 | 0.010 | 0.003 |
| **UBC809_493** | 0.14 | 0.51 | 0.15 | 0.94 | 0.118 | 0.552 | 0.096 | 0.070 | 0.042 | 0.616 | 0.439 | 0.083 | 0.115 | 0.544 | 0.036 | 0.059 | 0.036 | 0.551 | 0.356 | 0.047 |
| **UBC809_562** | 0.34 | 0.56 | 0.23 | 0.96 | 0.213 | 0.053 | 0.983 | 0.774 | 0.949 | 0.080 | 0.670 | 0.834 | 0.196 | 0.001 | 0.830 | 0.779 | 0.835 | 0.003 | 0.750 | 0.782 |
| **UBC881_320** | 0.16 | 0.50 | 0.13 | 0.91 | 0.696 | 0.042 | 0.889 | 0.906 | 0.966 | 0.919 | 0.535 | 0.805 | 0.426 | 0.003 | 0.857 | 0.627 | 0.843 | 0.643 | 0.165 | 0.563 |
| **UBC885_524** | 0.23 | 0.50 | 0.08 | 0.76 | 0.131 | 0.800 | 0.091 | 0.044 | 0.034 | 0.525 | 0.994 | 0.134 | 0.075 | 0.585 | 0.020 | 0.031 | 0.023 | 0.524 | 0.674 | 0.081 |
| **UBC885_537** | 0.14 | 0.50 | 0.08 | 0.84 | 0.844 | 0.992 | 0.982 | 0.783 | 0.861 | 0.313 | 0.907 | 0.957 | 0.597 | 0.627 | 0.713 | 0.785 | 0.823 | 0.050 | 0.112 | 0.900 |
| **UBC885_859** | 0.34 | 0.60 | 0.26 | 0.98 | 0.031 | 0.426 | 0.151 | 0.062 | 0.070 | 0.020 | 0.890 | 0.077 | 0.016 | 0.428 | 0.007 | 0.014 | 0.012 | 0.003 | 0.784 | 0.042 |
| **UBC888_485** | 0.48 | 0.75 | 0.32 | 1.00 | 0.663 | 0.623 | 0.612 | 0.372 | 0.378 | 0.507 | 0.918 | 0.626 | 0.125 | 0.027 | 0.004 | 0.015 | 0.007 | 0.403 | 0.172 | 0.146 |
| **UBT4_271** | 0.07 | 0.48 | 0.00 | 0.64 | 0.804 | 0.911 | 0.960 | 0.700 | 0.806 | 0.304 | 0.744 | 0.990 | 0.463 | 0.789 | 0.534 | 0.487 | 0.646 | 0.038 | 0.115 | 0.889 |
| **UBT4_321** | 0.09 | 0.49 | 0.03 | 0.84 | 0.962 | 0.978 | 0.947 | 0.878 | 0.970 | 0.364 | 0.839 | 0.864 | 0.915 | 0.805 | 0.917 | 0.958 | 0.928 | 0.112 | 0.281 | 0.498 |
| **UBT4_674** | 0.16 | 0.50 | 0.06 | 0.78 | 0.501 | 0.979 | 0.682 | 0.446 | 0.458 | 0.243 | 0.953 | 0.537 | 0.054 | 0.720 | 0.093 | 0.109 | 0.104 | 0.023 | 0.660 | 0.127 |
